# Acute and Chronic Cerebral Hypoperfusion After Flow-Diverter Placement: Is There a Role for Extra–Intracranial Bypass? A Case Series and Narrative Literature Review

**DOI:** 10.3390/neurolint18080151

**Published:** 2026-08-17

**Authors:** Alessandro Tozzi, Valentina Caldiera, Giuseppe Ganci, Paolo Ferroli, Marco Schiariti, Matteo Milani, Isabella Canavero, Benedetta Storti, Anna Bersano, Francesco Acerbi, Elisa Francesca Maria Ciceri

**Affiliations:** 1 Interventional Neuroradiology Unit, Neurosurgery Department, Fondazione IRCCS Istituto Neurologico “Carlo Besta”, 20133 Milan, Italy; 2 Department of Clinical Sciences and Community Health, University of Milan, 20122 Milan, Italy; 3 Unit of Neurosurgery, Neurosurgery Department, Fondazione IRCCS Istituto Neurologico “Carlo Besta”, 20133 Milan, Italy; 4 Cerebrovascular Unit, Department of Clinical Neurosciences, Fondazione IRCCS Istituto Neurologico “Carlo Besta”, 20133 Milan, Italy; 5 Department of Translational Research and New Technologies in Medicine and Surgery, University of Pisa, 56124 Pisa, Italy; 6 Unit of Neurosurgery, University Hospital of Pisa, 56124 Pisa, Italy

**Keywords:** extracranial–intracranial bypass, flow diverter, aneurysm, in-stent thrombosis, STA-MCA bypass

## Abstract

**Background:** Flow diverters (FDs) are widely used to treat intracranial aneurysms. Although effective, FD-related thrombosis or progressive stenosis may lead to acute or delayed cerebral hypoperfusion with significant neurological morbidity. Management options are limited when medical or endovascular rescue strategies fail. This study aims to evaluate the role of extracranial–intracranial bypass (EC-IC bypass) as a rescue strategy across the spectrum of FD-related cerebral hypoperfusion. **Methods:** We retrospectively reviewed all patients who underwent EC-IC bypass for FD-related cerebral hypoperfusion over a 10-year period in our institution. Both acute and chronic presentations were included. Clinical status was assessed using the modified Rankin Scale (mRS). Imaging evaluation included digital subtraction angiography (DSA), computed tomography angiography (CTA), magnetic resonance angiography (MRA) and quantitative magnetic resonance imaging for bypass flow assessment. Surgical technique, antiplatelet therapy, timing of intervention and clinical outcomes were analysed. **Results:** Five patients with FD-related cerebral hypoperfusion who underwent superficial temporal artery–middle cerebral artery bypass (STA-MCA bypass) were identified. Two distinct patterns emerged. Three patients developed early hypoperfusion within the first month of FD placement, presenting with acute neurological deficits and treated by emergent or urgent bypass under dual antiplatelet therapy. Two patients developed late hypoperfusion years after the procedure, presenting with progressive haemodynamic symptoms and treated by elective flow-augmentation bypass under aspirin monotherapy. At discharge, only one patient had an mRS ≥ 3. At one-year follow-up, a favourable outcome (mRS ≤ 2) was observed in all patients with available data. Follow-up data were missing for one patient. **Conclusions:** EC-IC bypass represents a feasible rescue strategy for FD-related cerebral hypoperfusion when medical or endovascular treatments fail. In our experience, though limited, STA-MCA bypass can provide sufficient cerebral reperfusion, even in urgent settings and under dual antiplatelet therapy, leading to favourable neurological outcomes.

## 1. Introduction

Currently, Flow Diverters (FDs) represent the main endovascular strategy for the treatment of a wide range of intracranial aneurysms. By redirecting blood flow away from the aneurysmal sac, FDs promote intrasaccular thrombosis (of the aneurysmal sac) while providing a scaffold for endothelial healing of the parent vessel [1,2,3]. Ischaemic complications remain among the most feared events during and after endovascular treatment. Despite technical advances and widespread use of dual antiplatelet therapy, thrombosis of the device may still occur and, if not rapidly addressed, often results in large territorial infarction [3,4,5]. In acute cases, standard medical rescue measures include intravenous or intra-arterial glycoprotein IIb/IIIa inhibitors (e.g., Tirofiban-Aggrastat^®^, Medicure International Inc., 1st Floor Limegrove Centre, Holetown, St. James, Barbados), sometimes combined with mechanical thrombectomy (MT) or percutaneous transluminal angioplasty (PTA) [3,6]. Stent retrieval is often technically demanding, is not always successful and carries a risk of further complications. However, the stented vessel may progressively become occluded or severely stenotic, leading to various degrees and manifestations of cerebral hypoperfusion that can develop at different times from intervention. In these scenarios, surgical revascularization may represent a possible therapeutic option. Extracranial–intracranial bypass (EC-IC bypass) has traditionally been reserved for elective settings and chronic states of cerebral hypoperfusion [7]. However, its role as a rescue therapy for iatrogenic acute and chronic hypoperfusion following intravascular device placement has been poorly documented and is largely confined to isolated reports [2,8,9].

This study aims to describe the role of EC-IC bypass as a rescue flow-augmentation strategy across the spectrum of flow diverter-related cerebral hypoperfusion by presenting a case series of five patients who underwent urgent or elective EC-IC bypass following FD placement.

## 2. Materials and Methods

A retrospective review of the endovascular and neurosurgical databases of our institution was conducted (September 2015–September 2025) to identify cases of flow diverter-related complications managed with rescue EC-IC bypass. Both acute and chronic occlusions were included, regardless of whether the flow diverter had been implanted at the Authors’ institution or at referring centres.

Data collected included demographic information, underlying pathologies leading to stent placement, potential causes of occlusion and technical complications during deployment, timing of occlusion, antiplatelet therapy at the time of FD placement and bypass, revascularization techniques and clinical status at the time of flow diverter placement, bypass surgery, hospital discharge and at one year (Table 1).

Clinical assessment was performed pre- and postoperatively by an experienced vascular neurologist, and the modified Rankin Scale (mRS) was utilized to assess clinical status [10]. Neuroimaging assessment included: digital subtraction angiography (DSA), dual-energy computed tomography angiography (DE-CTA), magnetic resonance imaging (MRI) including magnetic resonance angiography (MRA, TOF sequence), and quantitative MRI (qMRI) using the NOVA^®^-Noninvasive Optimal Vessel Analysis software (VasSol, 348 Lathrop Ave, River Forest, IL, USA) for flow assessment at the bypass site.

In all cases, the indication for surgical revascularization was established by a multidisciplinary vascular board including interventional neuroradiologists, vascular neurosurgeons and vascular neurologists. EC-IC bypass was considered only after medical and/or endovascular rescue strategies had already failed or when further endovascular manipulation was judged to carry an unacceptable risk. The decision was based on the following domains:

- Clinical status: presence of fluctuating or progressive neurological deficits, or deficits refractory to blood-pressure augmentation.
- Perfusion CT imaging: reduced cerebral blood flow (CBF) and prolonged mean transit time (MTT) in the affected territory on computed tomography perfusion (CTP).
- MRI: areas of restricted diffusion in the brain parenchyma.
- Status of the parent vessel and of the device: degree of in-stent thrombosis or stenosis, or device modification, i.e., shrinkage, malapposition or migration.
- Estimated risk and benefit of endovascular retreatment: thromboembolic risk of repeated manipulation within a thrombosed or migrated device, risk of further device deformation or dislodgement, expected efficacy of the manoeuvre and patency after re-intervention.

For the purpose of this study, FD-related hypoperfusion was characterized along two dimensions. The interval between FD placement and the hypoperfusion event was defined as *early* when it occurred within the first month (acute, <24 h; subacute, 1–30 days) and *late* when it occurred more than 30 days after the procedure. The mode of clinical presentation was defined as *acute* when neurological deficits developed abruptly and *progressive* when symptoms of haemodynamic insufficiency developed gradually over weeks to months. Accordingly, bypass was performed on an emergent or urgent basis in patients with acute presentation and a clinical–radiological mismatch suggesting salvageable tissue, with the aim of preventing infarct progression, and on an elective basis in patients with progressive presentation, in whom the parent vessel occlusion was considered irreversible and the aim of surgery was chronic flow augmentation.

To provide a broad overview of the current state of the art in the management of FD-related haemodynamic complications, a narrative literature review was conducted using PubMed. The search was limited to studies published between January 2000 and January 2026 and was performed using the keywords “flow-diverter AND (occlusion OR thrombosis OR bypass OR stentectomy OR angioplasty OR thrombectomy).” Studies reporting all the available rescue techniques for hypoperfusion-related complications occurring in the acute or subacute setting after FD placement are included in Table 2. An additional PubMed search was conducted to specifically investigate the role of EC-IC bypass as a rescue strategy over the same period and with the same language restriction, using the keywords “bypass AND (urgent OR emergency OR flow-diverter OR stent).” Articles describing emergency bypass procedures performed for complications of endovascular treatment, ischaemic stroke, or iatrogenic intracranial vascular injury are included in Table 3. Articles were identified and screened for relevance to the predefined topics by two authors (A.T. and M.M.). Duplicate records, studies not directly addressing the selected clinical scenarios and articles published in languages other than English were excluded. 

Given the narrative nature of the review, no formal systematic-review protocol, PRISMA flow diagram, or risk-of-bias assessment was performed.

Given the retrospective design, the Ethics Committee waived study registration and the requirement for written informed consent for participation. Reporting followed the STROBE guidelines for cohort studies. Written informed consent for the publication of anonymised clinical data and images was obtained from the patient reported as an illustrative case.

## 3. Results

The results are summarized in Table 1. During the study period five patients who underwent EC-IC bypass for flow diverter-related cerebral hypoperfusion were identified. In two cases (patients 1 and 2), the endovascular procedure was performed at our institution, whereas in the remaining three cases (patients 3–5) were referred after FD placement elsewhere. In all cases pre- and postoperative DSA, DE-CTA, and MRA were performed. All the endovascular procedures had been performed for left internal carotid artery (ICA) aneurysms. In four patients malpositioning, shrinkage, incomplete opening or migration was reported.

Two distinct patterns of FD-related hypoperfusion were identified. In three patients (cases 1–3), hypoperfusion occurred within the first month of the procedure and presented with abrupt neurological deterioration. Acute in-stent thrombosis was initially managed with intravenous tirofiban in cases 1 and 2, combined with MT in case 2, whereas case 3 developed subacute thrombosis three days after FD placement and was treated by MT alone. Despite initial angiographic success in cases 1 and 2, recurrent in-stent thrombosis with symptomatic hypoperfusion developed within days in case 1 and one month later in case 2, while in case 3 vessel thrombosis persisted despite endovascular rescue treatment. In the remaining two patients (cases 4 and 5), hypoperfusion developed years after the endovascular procedure and followed a progressive course, with symptoms of haemodynamic insufficiency evolving gradually rather than abruptly. Case 4 showed ICA apex stenosis due to upward stent migration three years after FD placement, and case 5 showed progressive occlusion of the stented ICA five years after implantation; in both, the parent vessel occlusion was considered irreversible and no longer compensated by collateral circulation.

All patients were treated by superficial temporal artery-middle cerebral artery single-barrel bypass (STA-MCA bypass) using the standard technique [11]. Three patients (cases 1, 2, and 3) underwent revascularization while receiving dual antiplatelet therapy (DAPT). In one patient (case 1), surgery was performed without antiplatelet washout, whereas in the remaining two patients a short clopidogrel washout was implemented (5 and 3 days, respectively). The remaining two patients (cases 4 and 5) were receiving aspirin monotherapy (single antiplatelet therapy—SAPT) at the time of surgery, as dual antiplatelet therapy had been discontinued earlier in the clinical course. In case 1 no antiplatelet agent was interrupted. In the remaining patients the dose of aspirin was omitted on the day of surgery and resumed the following day, as is standard practice in our centre for all revascularization procedures; this does not constitute a washout, since inhibition of platelet cyclo-oxygenase is irreversible for the lifespan of the circulating platelets. No surgical or systemic complications were observed, in particular no haemorrhage, infection or thromboembolic event.

In three patients (case 1, 3 and 4), a NOVA^®^ study was performed only after surgery, showing immediate postoperative bypass flows ranging from 46 to 101 mL/min. Sampling was performed on postoperative day (POD) 3 for case 1, POD 2 for case 3 and POD 5 for case 4. NOVA^®^ data are not available for patients 2 and 5, because the system was not available at the time of their admission. At baseline all the patients had an mRS of 0. In four cases, neurological deterioration was observed after the endovascular procedure, with two cases (cases 1 and 3) showing a worsening of more than 1 mRS point. At discharge after EC-IC bypass, only one patient showed an mRS ≥ 3. At 1-year follow-up, all the patients had an mRS ≤ 2; follow-up data were unavailable for case 3.

Over time, reopening of the parent vessel with closure of the bypass was observed in case 1. In the other cases (2, 4 and 5) the bypass remained patent throughout follow-up, with no recanalization of the occluded vessel. The data regarding bypass patency are not available for case 3. Case 3 had been referred from another institution and returned there after discharge; no further clinical or imaging follow-up was available.

Regarding the narrative review 24 studies met the inclusion criteria and were included in the final narrative synthesis (Table 2 and Table 3).

### ILLUSTRATIVE CASE—Patient 1

**Figure 1:**
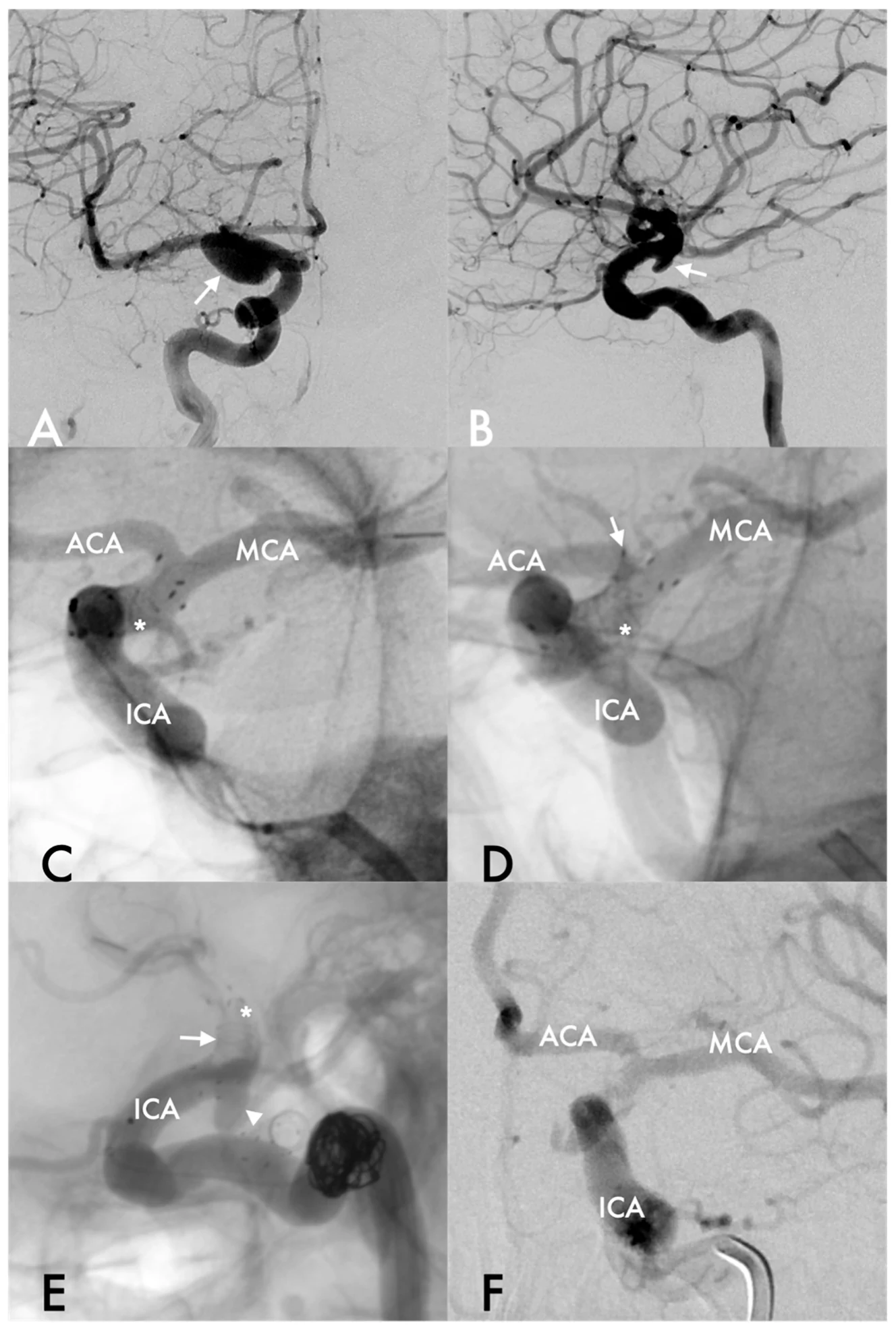
DSA shows the right ICA aneurysm (arrow) (**A**) and the left ICA aneurysm (**B**) in AP and LL projections, respectively. The FD (asterisk) is well positioned in the left ICA, with both the ACA and MCA well opacified (**C**). A few minutes later, the FD (asterisk) migrates cranially toward the bifurcation (arrow) (**D**). Follow-up series show poor opacification of the carotid apex (arrow) within the FD (asterisk), MCA, and ACA, while the aneurysm sac is clearly visible (arrowhead) (**E**,**F**). ACA: anterior cerebral artery; MCA: middle cerebral artery; ICA: internal carotid artery.

A middle-aged woman with a history of hypertension and a 40-pack-year smoking history was referred to our centre for an evaluation of two incidental mirror aneurysms of ICA at the level of the posterior communicating artery (PComA) (Figure 1A,B; Table 1). The right-sided aneurysm was treated first by means of flow diversion using a FRED stent (4 × 17 mm; MicroVention, Terumo Neuro, Aliso Viejo, CA, USA), combined with adjunctive coiling (Target 360 Soft; Stryker, Fremont, CA, USA), under DAPT (75 mg of clopidogrel and 100 mg of aspirin daily, initiated 5 days before the procedure). The endovascular procedure was uneventful, and the patient was discharged home without neurological deficits.

After five months, the left ICA aneurysm was treated under the same antiplatelet premedication. Via a bi-axial system (Envoy 6F and Headway-27 MicroVention, TerumoNeuro—Aliso Viejo, CA, USA; Hybrid 12–14 microguide, Balt Extrusion—Montmorency, France) the FD (FRED 4 × 18 MicroVention, TerumoNeuro—Aliso Viejo, CA, USA) was carefully deployed across the aneurysmal neck. Final angiographic control revealed cranial stent displacement with strut compaction and acute in-stent thrombosis (Figure 1C–F).

**Figure 2:**
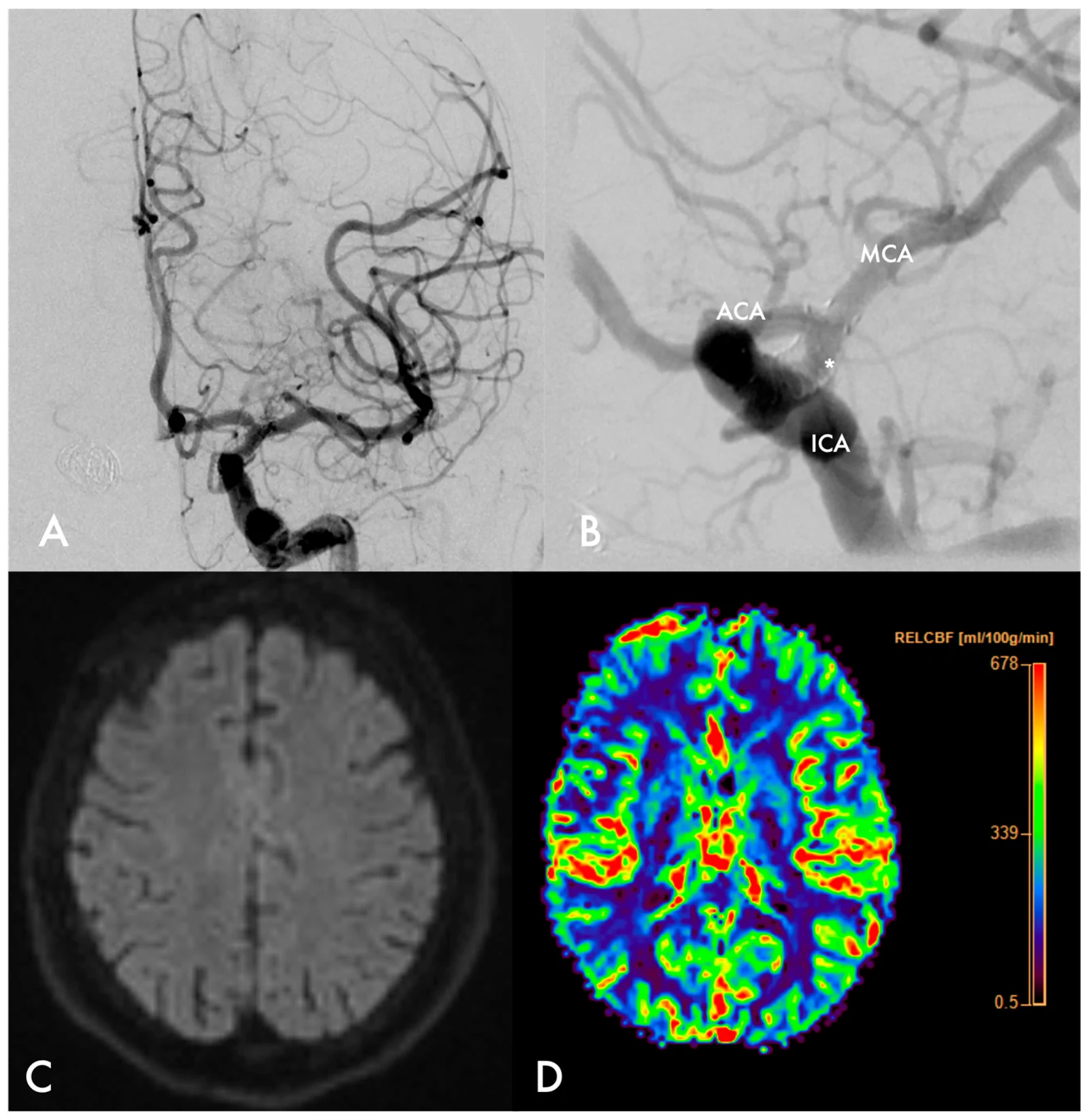
After administration of tirofiban, sufficient perfusion is achieved throughout the ICA, as well as in the ACA and MCA. The stent (asterisk) appears unchanged in position and patent (**A**,**B**). Follow-up MRI on postoperative day 2 shows no areas of restriction on diffusion-weighted sequences (**C**). The CTP study demonstrates normal CBF in the left hemisphere (**D**).

Tirofiban was immediately administered as an intravenous bolus followed by 18 h maintenance infusion, according to protocols (0.25 mcg/kg/min for 3 min followed by continuous infusion of 0.1 mcg/kg/min for 18 h). Serial DSA controls at 20, 40 and 60 min showed gradual recanalization of the occluded segment, with restoration of flow in the distal territories without significant slowdowns (Figure 2A,B). No infarcts were visible in the post-procedural MRI with normal diffusion-weighted imaging (DWI). The patient was transferred to the Neuro Intensive Care Unit (NICU) for blood pressure augmentation to support cerebral perfusion and neurological monitoring. Despite angiographic FD patency on POD 1, she developed transient ischaemic symptoms (reduced consciousness, aphasia and right hemiparesis) in the following days. MRI on POD 2 confirmed preserved cerebral perfusion without ischaemic lesions (Figure 2C,D). DAPT therapy was continued.

**Figure 3:**
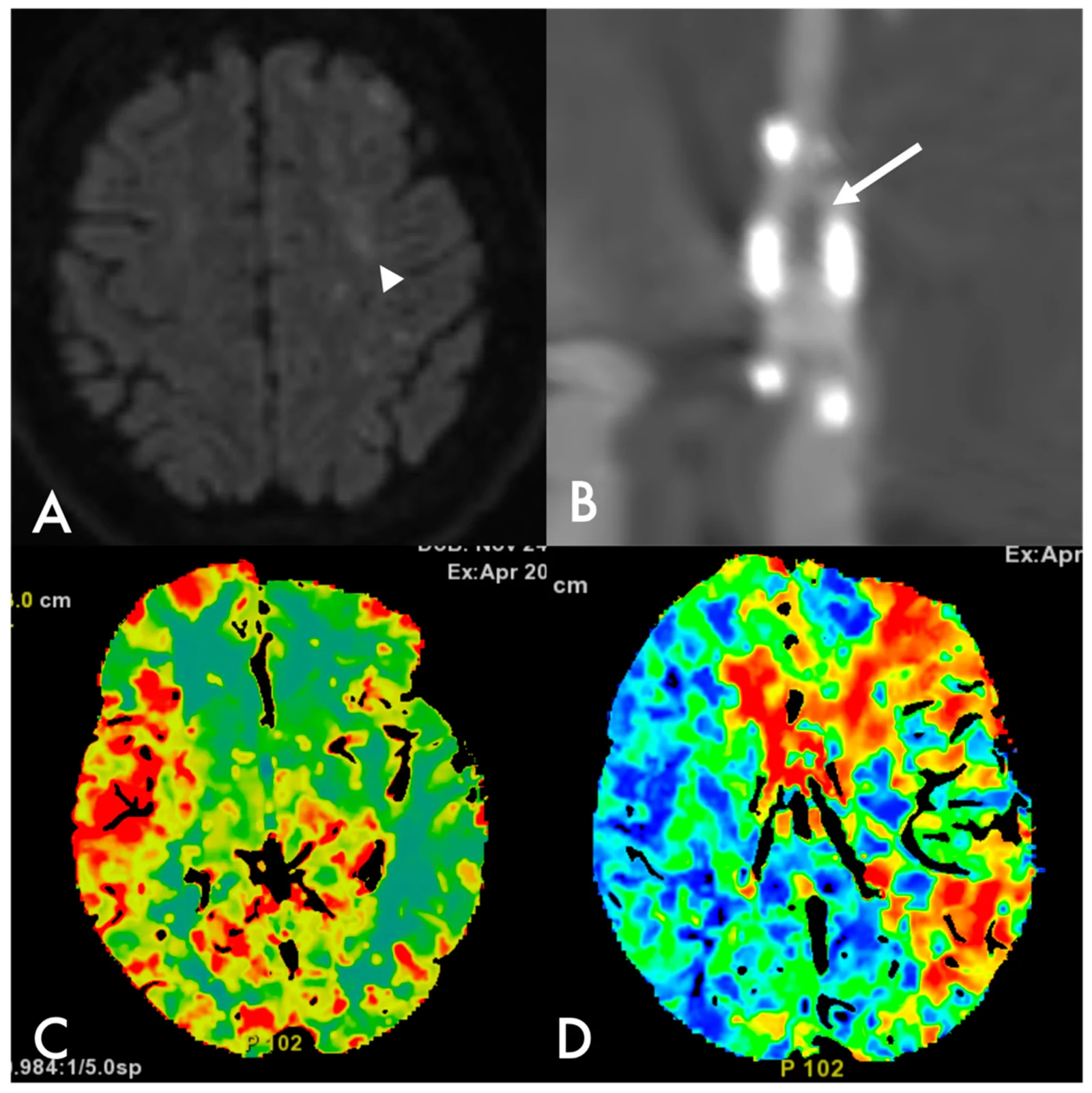
Diffusion MRI performed on postoperative day 5 shows areas of signal restriction compatible with recent ischemia (arrowhead) (**A**). DE-CTA with iodine map and stent reconstruction demonstrates thrombosis in its distal portion (arrow) (**B**). CTP maps show reduced CBF in the left hemisphere (**C**) and prolonged MTT in the watershed ACA-MCA territory (**D**).

**Figure 4:**
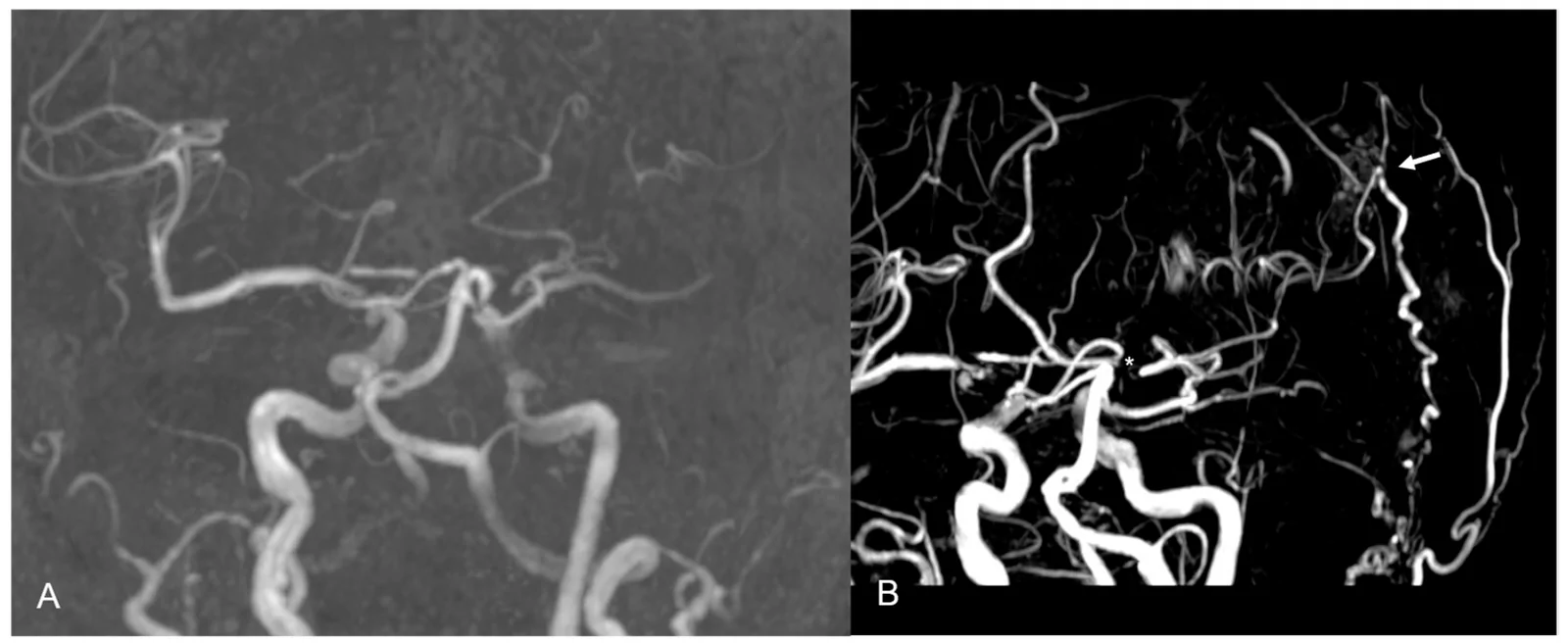
Pre-bypass MRA demonstrates poor visualization of the left MCA territory (**A**). Post-bypass MRA shows the result of the anastomosis (arrow) with better representation of the MCA circulation (the asterisk marks the flow diverter) (**B**).

On POD 5, the patient experienced sudden neurological deterioration, characterized by severe aphasia and right hemiplegia (mRS 5). Brain MRI revealed areas of restricted diffusion in the middle cerebral artery–anterior cerebral artery (MCA–ACA) watershed territories, with reduced visualization of MCA branches on MRA (Figure 3A and Figure 4A). The CTP scan showed reduced CBF and prolonged MTT, while the DE-CTA revealed recurrent ICA in-stent thrombosis and FD shrinkage (Figure 3B–D).

After vascular board discussion, further endovascular options (ICA occlusion, stent removal, or MT) were considered to carry an unacceptably high thromboembolic risk considering the poor collateralization from anterior cerebral artery, while surgical revascularization was favoured. An urgent STA-MCA bypass was therefore performed the same day the patient underwent DAPT, following transfusion of two units of platelets to lower the haemorrhagic risks [12]. The procedure was uneventful, with no significant blood loss or other complications.

**Figure 5:**
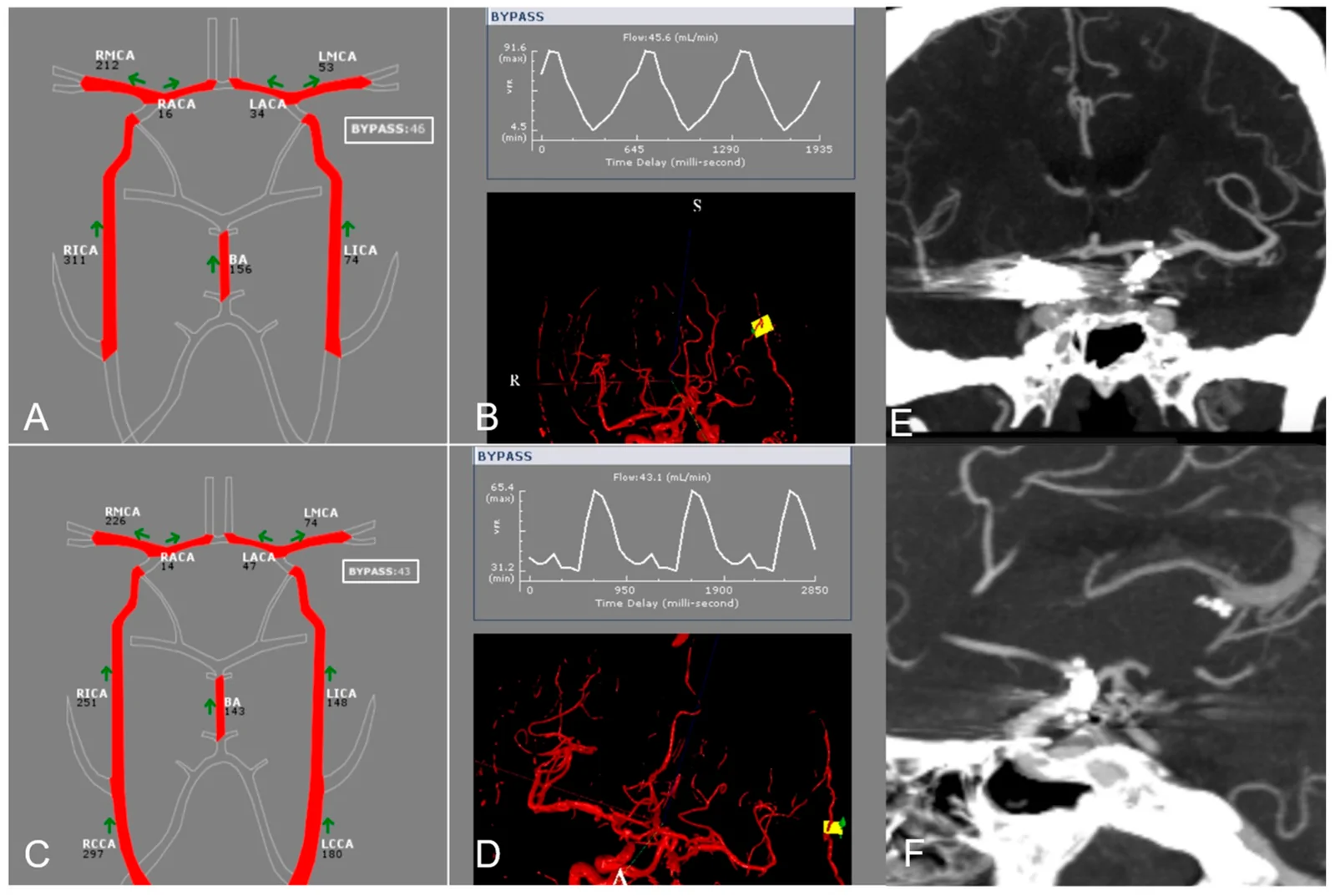
Postoperative qMRI NOVA^®^ demonstrated a flow of 53 mL/min in the MCA and 46 mL/min in the bypass (**A**,**B**). At the 7-day follow-up, NOVA^®^ showed stable bypass flow (46 vs. 43 mL/min) and a slight increase in MCA flow (53 vs. 74 mL/min) (**C**,**D**). The 4-year DE-CTA control showed complete exclusion of both aneurysms, patency of the FD and occlusion of the bypass, with restoration of physiological flow in the left MCA (**E**,**F**). RMCA: right middle cerebral artery; LMCA: left middle cerebral artery; RACA: right anterior cerebral artery; LACA: left anterior cerebral artery; BA: basilar artery; RICA: right internal carotid artery; LICA: left internal carotid artery; RCCA: right common carotid artery; LCCA: left common carotid artery.

Postoperative MRA confirmed bypass patency, while the NOVA^®^ study showed a flow of 53 mL/min in the MCA and 46 mL/min in the bypass (Figure 4B and Figure 5A,B). In the following days, the patient gradually improved, with progressive recovery from hemiparesis and resolution of aphasia. The NOVA^®^ study performed before discharge, on POD 7, showed stable flow in the bypass (46 vs. 43 mL/min) and an increase in MCA flow (53 vs. 74 mL/min), probably because flow through the previously thrombosed segment had already been restored (Figure 5C,D).

The patient was transferred to a rehabilitation facility with mild residual motor deficits (mRS 3). At the 3-month and one-year follow-up, she regained complete independence without any sensory-motor impairment (mRS 0).

The 4-year DE-CTA control demonstrated complete exclusion of both aneurysms with bilateral patency of the FD and bypass occlusion, with restoration of the physiologic flow in the left hemisphere (Figure 5E,F).

## 4. Discussion

Ischaemic stroke due to thromboembolism represents the most frequent perioperative and postoperative complication of FD implantation, with acute in-stent thrombosis rates of between 4 and 6% [3,13,14]. Immediate intervention is essential, as an untreated clot can rapidly enlarge, occluding both the FD and downstream vessels, and may also give rise to distal embolic showers, ultimately leading to severe ischaemic stroke [3,5].

During the 10-year study period, approximately 500 FDs were implanted at our institution. The rate of periprocedural FD occlusion was approximately 1.5%, with near-complete resolution in most cases following treatment with IIb/IIIa receptor antagonists. Our rate of complication is consistent with the recent literature [15,16]. Notably, only two of the patients who required a rescue bypass had been treated at our institution, which corresponds to about 0.4% of the flow-diverter procedures performed at our institution during the study period. The remaining three patients were referred after flow-diverter placement elsewhere and therefore do not contribute to this figure. These numbers show how rare this scenario is and how rescue STA-MCA bypass remains a niche procedure.

Various factors have been investigated to predict which patients are at higher risk for occlusion, including individual variability in response to antiplatelet therapy, patient compliance, potential drug interactions and comorbidities [3,17,18].

At our institution, since 2021, VerifyNow-P2Y12 testing has been routinely performed in patients receiving DAPT to assess platelet inhibition and identify rapid metabolizers or potential non-compliance [19]. This assay was not yet available at the time the first two cases were treated (cases 1–2). Nevertheless, adequate antiplatelet response and compliance can reasonably be assumed in case 1, as the patient had previously undergone contralateral FD implantation without complications. In this case, intra-stent thrombosis was most likely triggered by post-deployment FD shrinkage and displacement, potentially leading to endothelial injury. Smoking, which is the only patient-related factor consistently associated with post-stenting stroke, may have further contributed, although it is unlikely to fully explain the event [3]. Beyond patient-related factors, device malposition or migration appears to have played a central role, consistent with most reported cases of FD-related occlusion [8] (Table 1).

Several rescue strategies for intra-stent occlusion have been reported. The first-line approach for acute occlusion is the administration of IIb/IIIa receptor antagonists such as tirofiban, given intravenously or intra-arterially [3,6]. GPIIb/IIIa inhibitors work by binding to the specific receptors on the platelet surface, blocking platelet aggregation and reducing thrombus formation [4,6]. Other strategies include MT, PTA, stentectomy and EC-IC bypass (Table 2). While MT and PTA are more common procedures, bypass and stentectomy are usually reserved for highly experienced operators. Considering the few cases reported in the literature, stentectomy can be performed endovascularly with retrieval of the stent through an aspiration catheter together with a microsnare or microforceps-type device [20,21]. In cases 1 and 2 tirofiban was given in the acute phase and achieved transient recanalization, so stentectomy and other endovascular manoeuvres were not considered.

Notably, all recanalization manoeuvres carry a real procedural risk [15,22]. Mechanical manipulation of a thrombosed stent can fragment the clot and cause distal embolization, dissect or damage the device, injure the vessel wall, and, when antegrade flow is suddenly restored in a chronically hypoperfused territory, determine reperfusion injury [23]. Consistently, Nakajima et al. reported two patients with carotid stent thrombosis and impaired cerebrovascular reserve in whom STA-MCA bypass was deliberately preferred over recanalization for these reasons, achieving mRS 0 with no procedural complications and no recurrent ischaemic events at follow-up [23]. Our series includes two scenarios that differ in their pathophysiology. In the early occluded group, the device thromboses before any collateral supply has had time to develop, symptoms come on abruptly, and the tissue at risk is a true penumbra [24].

Here, the bypass must be performed within hours to a few days, and the aim is to stop the infarct from growing. In the late group, the in-stent stenosis or occlusion progresses over years, and collaterals have time to develop, so symptoms appear only once cerebrovascular reserve is exhausted. The parent vessel is irreversibly occluded and the aim of the bypass is no longer tissue salvage but chronic flow augmentation and stroke prevention [7,24,25]. Urgency, antiplatelet management and the expected outcome therefore differ between the two groups.

Examining the existing literature on EC-IC bypass revascularisation, most studies focus on patients with spontaneous ischaemic stroke, either thromboembolic or haemodynamic (Table 3). Only three papers report cerebral hypoperfusion due to FD occlusion treated with EC-IC bypass, one of which was in the acute setting [8,9,26].

Yi et al. described a subacute (8 days) device occlusion managed with an emergency EC-IC bypass under DAPT, achieving an excellent clinical outcome, as in our early-presentation cases. In that report, a high-flow bypass was chosen [8]. Lanterna et al. reported a symptomatic FD thrombosis treated three months after implantation with a double-barrel bypass and full neurological recovery [9]. Shoda et al. include bypass among the rescue procedures used for perioperative ischaemic events and report one patient who developed hemiplegia. Device type, patient characteristics, timing of the bypass and individual outcomes are not given separately, so that case cannot be compared directly with ours (Table 2) [26].

Of note, the case reported by Yi et al. involved an LVIS stent, which we considered comparable to a flow diverter given its metal coverage (23%), closer to that of standard FDs, such as the Pipeline (30%), than to conventional stents (8%) [27].

In our series we preferred the STA-MCA bypass because it is easier to manage in an emergency setting and carries a lower risk of perioperative complications [11]. Furthermore, in all our patients the haemodynamic impairment involved mostly the MCA territory; thus, an STA-MCA bypass could be considered sufficient to provide the flow augmentation needed to improve MCA circulation. In addition, bypass flow has been shown to adapt to the demands of the revascularized territory and can reach high values when required [28]. In fact, the postoperative NOVA^®^ study confirmed that the anastomosis provided adequate flow, ranging from 46 to 101 mL/min (Table 1).

Furthermore, in all patients neurological symptoms improved after the procedure, except for case no. 5, who experienced stability of the deficit (mRS 2 after the bypass and at 1-year follow-up). Over time, reopening of the parent vessel with gradual closure of the bypass was evident in case 1. We could hypothesize that STA-MCA bypass was useful to protect the patients from further vascular events in the early postoperative period by immediately improving regional blood flow, and that the subsequent occlusion was only related to the restoration of adequate and more physiological intra-stenting flow. On the contrary, the STA-MCA bypass usually remains patent when the indication is an irreversible stenosis or occlusion of the parent artery (cases 2, 4 and 5).

At bypass surgery three patients (case 1, 2, and 3) were on DAPT, while two patients (case 4 and 5) were on SAPT with aspirin alone. Based on our experience, it is preferable to allow for a few days to pass for at least one antiplatelet agent to be discontinued. However, if urgent revascularization is required, such as in case 1, surgery can still be performed after platelet infusion to lower any haemorrhagic risks [12].

Although this research represents a limited single-centre experience, our series points out that EC-IC bypass can be a reasonable option in selected cases of acute FD-related complications. Additionally, bypass may also be used for flow augmentation in chronic occlusions following endovascular procedures, as is commonly done in cerebrovascular steno-occlusive disease [7,29]. Based on our experience, it is important to emphasize that ongoing dual antiplatelet therapy should not discourage performing bypass surgery when indicated. This principle may also extend to other clinical scenarios beyond FD-related complications, such as persistent occlusion of a major intracranial vessel after stroke. To our knowledge, this is also the first report of an STA-MCA bypass performed as an emergency procedure under DAPT for an FD-related complication.

Several limitations must be acknowledged. The sample is small, and the cases are heterogeneous, differing in the timing of occlusion and treatment, the type of flow diverter, the centre where the procedure was performed and the medical therapy used. The focused literature review is narrative rather than systematic: despite predefined search terms and screening by two authors, relevant studies may have been missed, and our synthesis should not be read as exhaustive. Finally, by design, the series includes only patients treated by bypass: it describes a surgical experience of revascularization after failed or unfeasible endovascular management and is not intended to describe the incidence or the endovascular management of FD-related hypoperfusion.

## 5. Conclusions

Ischaemic complications of flow diverters are a potentially serious drawback of the endovascular treatment of cerebral aneurysms. Prompt recognition and intervention are essential, and urgent EC-IC bypass can be an emergency option performed even in patients on DAPT. In our experience, though limited, an STA-MCA bypass can provide sufficient cerebral reperfusion, allowing for neurological recovery, as observed in our series, both in urgent settings and in delayed chronic occlusion. Bypass should be considered in selected cases as a potential rescue strategy in case of haemodynamic insufficiency when medical therapy or endovascular treatment has failed.

## Figures and Tables

**Table 1:** This table provides an overview of our cases managed with EC-IC bypass for symptoms of hypoperfusion in patients previously treated with FDs. The following information was recorded: the indication for the endovascular procedure, any complications occurring during the endovascular procedure and corresponding attempts at acute resolution, the rationale for the bypass, the surgical revascularization technique and its timing, antiplatelet therapy at the time of surgery and any washout period, quantification of flow in the bypassed vessel using qMRI NOVA^®^, and the modified Rankin Scale (mRS) assessed at baseline (pre-endovascular procedure), immediately after FD placement, before bypass, at post-bypass discharge and at 1-year follow-up. FD: flow diverter; ICA: internal carotid artery; AComA: anterior communicating artery; PComA: posterior communicating artery; STA: superficial temporal artery; MCA: middle cerebral artery; FU: follow-up; MT: mechanical thrombectomy.

	Pathology	Endovascular Procedure Issues	Rationale for Bypass	Technique of Reperfusion and Timing	Antiplatelet at Surgery	Nova^®^ Bypass Flow	mRS
1	Female, 69 yo; left pComA aneurysm	FD shrinkage (FRED, MicroVention). Acute complete thrombosis initially resolved with tirofiban.	ICA occlusion not tolerable (absent AComA cross-flow); stentectomy/MT with high embolic risk within a shrunken device, already re-thrombosed after tirofiban rescue.	Emergent STA-MCA bypass (at time of symptoms and 5 days after FD placement)	Aspirin and clopidogrel without washout	46 mL/min	0 before FD 3 after FD 5 at bypass 3 at discharge after bypass 0 at 1 year FU
2	Male, 60 yo; left supraclinoid ICA large dissecting aneurysm	First FD, malpositioning (Phenox p64 HPC). Second FD, incomplete opening (Phenox p64 HPC). Acute thrombosis resolved with tirofiban and MT.	Two overlapping, malposed devices with recurrent thrombosis after initial recanalization; durable patency unlikely, due to high embolic risk.	Urgent STA-MCA bypass (5 days after symptom onset and 1 month after FD placement)	Aspirin and clopidogrel (5 days washout)	n/a	0 before FD 1 after FD 2 at bypass 1 at discharge after bypass 0 at 1 year FU
3	Female, 49 yo; left pComA aneurysm	FD and coil, good positioning. Subacute (after 3 days) complete thrombosis unsuccessfully treated with mechanical thrombectomy (other centre),	Recanalization already failed with MT; further attempts have high embolic risk and low expected success.	Urgent STA-MCA bypass (72 h after symptom onset and 6 days after FD placement)	Aspirin and clopidogrel (3 days washout)	101 mL/min	0 before FD 3 after FD 3 at bypass 2 at discharge after bypass n.a at 1 year FU
4	Male, 58 yo; left paraophthalmic segment of ICA aneurysm	First FD, malpositioning. Second FD, good positioning. Residual stenosis of ICA tip (other centre).	Chronic in-stent stenosis at the ICA apex; endovascular retreatment not indicated.	Non-urgent STA-MCA bypass (progressive symptoms 3 years after FD placement)	Aspirin	58 mL/min	0 before FD 0 after FD 1 at bypass 1 at discharge after bypass 0 at 1 year FU
5	Female, 40 yo; left paraophthalmic ICA aneurysm	FD malpositioning Delayed complete occlusion (other centre)	Chronic total occlusion with marked device displacement; endovascular recanalization not feasible.	Non-urgent STA-MCA bypass (progressive symptoms 5 years after FD placement)	Aspirin	n/a	0 before FD 1 after FD 2 at bypass 2 at discharge after bypass 2 at 1 year FU

**Table 2:** This table summarizes studies reporting rescue techniques for occluded flow diverters. It includes the study title, first author, year of publication, number of cases reported, type of stent used, vessels in which the stent was deployed, any cases of incorrect deployment, the rescue technique employed (EC-IC bypasses are highlighted in bold), timing of FD occlusion and rescue intervention, and clinical outcomes. In the series by Shoda et al. individual-level data are not reported separately, so the bypass case cannot be compared directly with the other reports. PED: Pipeline Embolization Device, LVIS: Low-Profile Visualized Intraluminal Support, FRED: Flow Re-Direction Endoluminal Device, ICA: internal carotid artery, MCA: middle cerebral artery, AComA: anterior communicating artery, VA: vertebral artery.

Title	Author	Year	No of Cases	Stent Type	Vessel	Successful Deployment	Technique	Timing of Occlusion	Clinical Outcome
Extraintracranial Bypass as a Rescue Therapy for Symptomatic Flow Diverter Thrombosis	Lanterna L.A.	2015	1	PED	ICA	Minimal narrowing at the level of the aneurysmal bulge	**Double STA-MCA low-flow bypass**	Late (3 months)	No neurological deficit
Mechanical Thrombectomy of Acutely Occluded Flow Diverters	Samaniego E.A.	2019	2	PED	ICA-MCA and ICA	Yes	Glycoprotein IIb/IIIa inhibitor and mechanical thrombectomy	Acute (1 h)	Mild hemiparesis
High-Flow Extracranial-to-Intracranial Bypass for Treatment of Thrombotic Parent Vessel Occlusion After Stent-Assisted Aneurysm Coiling as a Potential Rescue Therapy	Yi L.	2019	1	LVIS	ICA	Yes	Glycoprotein IIb/IIIa inhibitor and **ICA-ECA high flow bypass**	Subacute (8 days)	No neurological deficit
Endovascular management of acute postprocedural flow diverting stent thrombosis	Townsend R.K.	2019	10	Pipeline Flex and FRED	ICA and VA	1 of 10 stent kinking	Glycoprotein IIb/IIIa inhibitor and angioplasty with balloon	Acute (1 day)–Subacute (3 months)	A total of 2 of 10 cases with minor residual deficits
Mechanical Thrombectomy of Acutely Occluded Flow-Diverters–Neuroendovascular Surgical Technique Demonstration: 2-Dimensional Operative Video	Roa J.A.	2020	2	PED	ICA-MCA	Yes	Stentriever thrombectomy	Subacute (7 days)	No neurological deficit
Acute Intraprocedural Thrombosis After Flow Diverter Stent Implantation: Risk Factors and Relevance of Standard Observation Time for Early Detection and Management	Hohenstatt S.	2022	12	FRED, PED, P48, Silk, and Surpass	ICA, AComA, and MCA	Not reported	Glycoprotein IIb/IIIa inhibitor	Acute (<30 min)	No neurological deficit
Feasibility and Results of the Stentectomy Procedure Performed as Rescue Treatment for Acute Thrombosis of Self-Expandable Intracranial Stents: A Case Series	Barburoglu M.	2022	7	FRED, Silk, and Leo Baby	ICA and MCA	Not reported	Glycoprotein IIb/IIIa inhibitor and stentectomy	Acute (<30 min)–Subacute (10 days)	A total of 2 of 7 cases had minor residual deficits
Prediction of perioperative ischemic events in flow diverter implantation using the adenosine diphosphate-induced platelet aggregation level system	Shoda K.	2025	47	Pipeline, Shield, FRED, and Surpass	Not reported	Not reported	Glycoprotein IIb/IIIa inhibitor, mechanical thrombectomy, parent vessel occlusion, and **STA-MCA low-flow bypass**	Not reported	For the bypass patient: severe hemiplegia

**Table 3:** This table summarizes the recent literature on urgent EC-IC bypass procedures. It includes the study title, first author, year of publication, clinical indication for the bypass (e.g., stroke or surgical complication), number of cases reported, type of bypass (high flow with graft or low flow), any preprocedural anticoagulant or antiplatelet therapy and neurological clinical outcomes.

Title	First Author	Year	Clinical Indication	No of Cases	Type	Antiaggregant/Anticoagulant	Clinical Outcome
Ischemic stroke due to dissection of intracranial internal carotid artery: implications for early surgical treatment	Oka F.	2008	Spontaneous ICA dissection	2	Low flow	Anticoagulant	A total of 1 of 2 cases with complete recovery
Emergency extracranial-intracranial bypass surgery for acute ischemic stroke	Nussbaum E.S.	2010	Ischemic stroke	13	Low flow	Heparin and aspirin	Variable; improvement
Emergency In Situ Bypass during Middle Cerebral Artery aneurysm Surgery: Middle Cerebral Artery-Superficial Temporal Artery Interposition Graft-Middle Cerebral Artery Anastomosis	Jung J.	2012	Rupture of MCA aneurysm during clipping	1	Low flow	None	Complete recovery
Emergency EC-IC bypass for symptomatic atherosclerotic ischemic stroke	Horiuchi T.	2013	Ischemic stroke	58	Low flow	None	Variable; improvement
Urgent Extracranial-Intracranial Bypass in the Treatment of Acute Hemodynamic Ischemic Stroke: Case Report	Mracek J.	2013	Ischemic stroke	1	Low flow	None	Complete recovery
Urgent Cerebral Revascularization Bypass Surgery for Iatrogenic Skull Base Internal Carotid Artery Injury	Rangel-Castilla L.	2014	Iatrogenic ICA injury during skull base surgery	8	High flow	None	Variable; improvement
Emergent surgical embolectomy in conjunction with cervical internal carotid ligation and superficial temporal artery-middle cerebral artery bypass to treat acute tandem internal carotid and middle cerebral artery occlusion due to cervical internal carotid artery dissection	Inoue T.	2015	Spontaneous ICA dissection	1	Low flow	None	Complete recovery
Emergency Extracranial-Intracranial Bypass during Surgery for Anterior Clinoid Process Hemangiopericytoma with ICA Invasion—Case Report	Chen Y.	2015	Iatrogenic ICA injury during skull base surgery	1	High flow	Heparin during surgery	Dysarthria
Urgent Bypass Surgery Following Failed Endovascular Treatment in Acute Symptomatic Stroke Patient With MCA Occlusion	Lee C.Y.	2017	Ischemic stroke	1	Low flow	None	Complete recovery
Emergency extracranial-intracranial bypass to revascularize salvageable brain tissue in acute ischemic stroke patients	Burkhardt J.K.	2018	Ischemic stroke	8	Low flow	DAPT or aspirin	Variable; improvement
Safety and clinical outcomes of urgent superficial temporal artery-middle cerebral artery bypass—a single-institution retrospective analysis	Kimura T.	2020	Ischemic stroke	35	Low flow	Aspirin or clopidogrel	Variable; improvement
Efficacy and Safety of Emergency Extracranial-Intracranial Bypass for Revascularization within 24 Hours in Resolving Large Artery Occlusion with Intracranial Stenosis	Noh Y.H.	2021	Ischemic stroke	10	Low flow	None	Variable; improvement
Emergency extra-intracranial bypass surgery in a patient with neurologic deficit after an accident in carotid occlusive test: A case report	Ngo H.M	2022	ICA occlusion during BTO	1	Low flow	None	Complete recovery
Quantitative radiological analysis and clinical outcomes of urgent EC-IC bypass for hemodynamic compromised patients with acute ischemic stroke	Jo H.	2022	Ischemic stroke	41	Low flow	Not reported	Variable; improvement
Emergent microsurgical intervention for acute stroke after mechanical thrombectomy failure: a prospective study	Fiedler J.	2022	Ischemic stroke	3	Low flow	None	Variable; improvement
Revascularization with superficial temporal artery–middle cerebral artery anastomosis in spontaneous intracranial internal carotid artery dissection: illustrative case	Nounaka Y.	2024	Spontaneous ICA dissection	1	Low flow	DAPT	Complete recovery

## Data Availability

The data presented in this study are available on request from the corresponding author. The data are not publicly available due to privacy and ethical restrictions.

## Abbreviations

The following abbreviations are used in this manuscript.

ACA	Anterior cerebral artery
AComA	Anterior communicating artery
CBF	Cerebral blood flow
CTP	Computed tomography perfusion
DAPT	Dual antiplatelet therapy
DE-CTA	Dual-energy computed tomography angiography
DSA	Digital subtraction angiography
DWI	Diffusion-weighted imaging
EC-IC	Extracranial–intracranial
FD	Flow diverter
FU	Follow-up
ICA	Internal carotid artery
MCA	Middle cerebral artery
MRA	Magnetic resonance angiography
MRI	Magnetic resonance imaging
mRS	Modified Rankin Scale
MT	Mechanical thrombectomy
MTT	Mean transit time
NICU	Neuro Intensive Care Unit
NOVA^®^	Noninvasive Optimal Vessel Analysis
PComA	Posterior communicating artery
POD	Postoperative day
PTA	Percutaneous transluminal angioplasty
qMRI	Quantitative magnetic resonance imaging
